# Notes and Notices

**Published:** 1870-12

**Authors:** 


### NOTES AND NOTICES.

"Modern Women, and What is said of Them," being a reprint of a series of articles in the *Saturday Review.* Second series, published by J. S. Redfield, $2.00. 404 pp., 12 mo. Among the chapters are, Fashionable Women, Old Girls, Weak Sisters,

Grim Females, Widows, Dolls, Charming Women, Bored Husbands, Chaperons, First Lovê, Sweet Seventeen, Popular Women, Pretty Women, Men's Favorites, &c. &c. The subjects are tartly handled with the pen of a ready writer, and will be read with a great deal of interest, by both men and women, with a great diversity of opinion as to the points taken.

"LONDON LANCET"—A monthly Journal of British and Foreign Medicines, Physiology, Surgery, Chemistry, Criticism, and general Medical Literature and News—$5.00 a year, six copies Twenty Dollars—Wm. C. Herald, Agent, 52 John Street, New York.

"WOMEN."—The American Tract Society, 150 Nassau Street, New York, have issued two timely volumes, "Woman, her Dignity and Sphere," by a Lady. 303 pp., 75 cents. Truthfulness, Honor, Home Duties, What to Read, Friends we Make, School Life Habit, Conversation, Dress, Amusements, Religious Work, &c. A most appropriate present for every girl. Also, "The Young Lady's Guide," 468 pp., 12 mo., an elegant volume, with admirable articles, by SARAH TYTLER. "JOHN HALIFAX," Gent. Woman's Thoughts about Women, Fashion, Novel Reading, Love and Courtship, Before and after Marriage. Mrs. HANNAH MOORE'S Thoughts on Conversation, Female Knowledge, and Public Amusements, Social Position, and Culture due to Women, by Rev. Dr. WILLIAM R. WILLIAMS. "Education of the Heart, Woman's best Work," by Mrs. SARAH STICKNEY ELLIS—and "The Influence of Christianity on the Condition of Women," by JOHN ANGELL JAMES—all in one Two Dollar volume. It is one of the very best books published in ten years for our daughters.

"Earth Closets, and Earth Sewage," by GEORGE E. WARRING, Jr., (of Ogden Farm) ; published by the *Tribune* Association, for Fifty Cents, 154 Nassau Street, Ne\ York. This subject of utilizing all wastes is one of the most important which can engage human attention, as to mere material interests. The wastes of a man's body in one year will fructify an acre of ground, and will make a hundred blades of grass grow where one did not grow before ; and in the rapidly increasing population of the earth, and the increasing necessity of agricultural products, it becomes the duty of every intelligent mind to give critical attention to this most important subject of "Earth Sewage."

